# Using an urban child health index to detect intra-urban disparities in Sweden

**DOI:** 10.1177/1403494820980261

**Published:** 2020-12-18

**Authors:** Per Kåks, Mats Målqvist

**Affiliations:** Uppsala Global Health Research on Implementation and Sustainability (UGHRIS), Department of Women’s and Children’s Health, Uppsala University, Sweden

**Keywords:** Child health, index, Sweden, socio-economic factors, urban health, Child Health Services

## Abstract

**Aims:**

Children’s health is affected by the environment in which they live and grow. Within Sweden’s urban areas, several city districts can be classified as socio-economically disadvantaged. This article describes the creation of a child health index to visualise disparities within and between Sweden’s three major cities, and how these relate to indicators of demography and socio-economic status.

**Methods:**

Data were collected for seven child health indicators and seven socio-economic and demographic indicators from the Swedish Pregnancy Register, Child Health Services and Statistics Sweden. An index was created from the health indicators using principal component analysis, generating weights for each indicator. Correlations between index outcomes and socio-economic and demographic indicators were analysed using linear regression.

**Results:**

The largest variance in index values could be seen in Stockholm followed by Malmö, and the poorest mean index outcome was seen in Malmö followed by Gothenburg. The largest intra-urban percentage range in health indicators could be seen for tobacco exposure at 0–4 weeks (0.8–33.9%, standard deviation (*SD*)=8.8%) and, for the socio-economic and demographic indicators, foreign background (19.9–88.5%, *SD*=19.8%). In the multivariate analysis, index outcomes correlated most strongly with foreign background (*R*^2^=0.364, *p*=0.001).

**Conclusions:**

Children’s health follows a social gradient and a pattern of ethnic segregation in Swedish cities, where it can be visualised using an index of child health. The resulting map highlights the geographical distribution of these disparities, and displays in which city districts child health interventions may be most needed.

## Introduction

Tackling inequities in child health outcomes is a global priority. In order to accomplish equality in health for children, it is fundamental first to establish the magnitude of these disparities.

When the World Health Organization Commission on the Social Determinants of Health published its final report in 2008, it confirmed that social disparities are a main driver for poor health outcomes and survival in low-, middle- and high-income countries alike [1]. Such inequities can be seen not only as inter-regional differences or as rural-urban gradients, but also as distinct disparities within cities themselves. A pattern of larger differences in social conditions *within* urban regions rather than *between* regions can be seen in countries with vastly different social contexts [2]. With this in mind, it becomes clear that statistics on both child health and its social determinants must be broken down on a subregional level if these trends in geographic distribution are to be properly understood.

The relationship between socio-economic status and health status is not only detectable when comparing the lowest- and highest-ranking geographical areas or income groups on the ladder. Rather, the differences in health typically follow socio-economic belonging along a continuous course, where a lower position in the hierarchy can predict worse health regardless of where you start counting. This predictable pattern has been labelled the ‘social gradient of health’, and it permeates all cultural and economic contexts [1]. A growing body of evidence points towards discrepancies in *relative* socio-economic status within societies being fundamental for the effects of the social gradient on the health of a population [3]. As the early years in a person’s life are formative in both a positive and a negative sense, it is possible for a suboptimal socio-economic environment in childhood to have long-lasting effects on health and behaviour that are not necessarily mitigated by socio-economic mobility later in life [4].

Previous research in high-income countries has among other things shown a relationship between socio-economic adversity and risk of giving birth to babies that are preterm and have a low birth weight [5,6], lower rates of breastfeeding initiation and shorter duration [7] as well as more exposure to tobacco smoke during the infant period [8]. Similar trends can be seen later in childhood. Both child obesity and poor dental health are significantly correlated with socio-economic deprivation in countries that are otherwise well off [9,10].

The detrimental effects of unequal societies extend beyond somatic health outcomes. Children growing up in families with a lower income tend to have lower self-esteem and life satisfaction [11], and children growing up in areas with high income inequality are more likely to experience bullying and school violence [12,13].

Apart from a stable income, education and safe surroundings, the prevention and mitigation of poor health outcomes also require the ability to navigate the health system and knowledge of where to find help and support when needed. Some research hints towards community trust being an important factor for public health [14] which may explain a link between successful social integration in areas with high immigration and improvements in child health outcomes. Within Swedish metropolitan areas, there are a number of city districts that are classified as especially vulnerable areas. A lack of social integration in these areas has resulted in high rates of unemployment and unfinished high school education and subsequently lower socio-economic status than the national average [15,16].

Previous studies have set out to map how child health outcomes differ geographically in Sweden. A report published by the Nordic School of Public Health in 2013 presented an index for child health for the city of Gothenburg using unweighted indicators to highlight the social gradient [17]. The index was later developed to determine differences in child health between Sweden’s 290 municipalities [18]. The most thorough attempt to map intra-urban inequities in health in Sweden so far is the work of the Malmö Commission, which published its final report in 2013 [19]. The report provided an overview of how health outcomes and their determinants differed between areas in Malmö, Sweden’s third largest city, and put forth a road map for social sustainability to bridge these gaps. Such initiatives have provided baseline data that have offered guidance for decisions on geographical and thematic focus when designing health interventions. To put local initiatives in a broader context, it may, however, be useful to highlight disparities both within and between urban areas.

In the attempt to manage the impact of the social gradient on children’s well-being, disparities in child health can be clarified by breaking down statistical differences for several types of child health indicators on a subregional level in order to provide a baseline for future interventions. This article aims to visualise the current state of inequity in the health of young children from the in utero period up to the age of five in Sweden’s three major cities through the creation of a child health index, and to explore how disparities in index outcomes relate to indicators of demography and socio-economic status.

## Methods

### Indicator data collection

A review of available data sets was undertaken to identify potential indicators of child health in Sweden. Building on previous assessments of indicators of child health in Europe [18,20], the data collection strove ideally to cover several domains of young children’s health and well-being: (a) health outcomes – mortality, morbidity, injuries and mental health; (b) risk factors – exposure to tobacco and air pollution, and low birth weight; (c) protective factors – breastfeeding, physical activity, immunisation and parental support; and (d) access and utilisation of care and support – preschool enrolment and participation in parental support groups.

The criteria set for potential indicators were: evidence of relevance for children’s health and development, availability in national registers or through administrative authorities and repeated and standardised measurements in the relevant geographical areas. Furthermore, the indicators were chosen to represent different stages of early child development. Due to a lack of availability and standardisation of measurements of several indicators available from regional Child Health Services and national registers, the selection of indicators was narrowed by necessity. Seven health indicators were identified, which have been highlighted in earlier literature as relevant for children’s health [21,22], along with seven indicators of demography and socio-economic status.

The life stages and domains covered by the identified health indicators are presented in Table I. Data for two indicators of child health were collected from the Swedish Pregnancy Register: the share of children born during 2017 exposed to tobacco in utero through maternal tobacco use during gestational weeks 30–32, and the prevalence of birth weight <2500 grams during the same year. Through matching with individual data on parental housing locality through Statistics Sweden, indicator data were calculated for each individual city district.

**Table I.** Health indicators and corresponding life stages.

Indicator	Domain	In utero	Birth	Neonatal period (0–1 month)	Infant period (1–12 months)	Early childhood (1–5 years)
Tobacco exposure in utero	Risk factor	✓				
Low birthweight	Risk factor		✓			
Tobacco exposure at 0–4 weeks	Risk factor			✓		
Breastfeeding at four months	Protective factor				✓	
Enrolment in day care or pedagogic care from one to five years	Utilisation of support					✓
Vaccination for MMR at two years	Protective factor					✓
Overweight or obesity at four years	Health outcome					✓

MMR: measles, mumps and rubella.

Data on the share of children aged one to five years living in each city district enrolled in day care or pedagogic care were derived from Statistics Sweden. These statistics represent prevalence on 31 December 2017.

Data for the other health indicators were collected from the regional Child Health Services. These data were geographically aggregated from child welfare centres located in the respective city districts during 2017, representing where the care was delivered. Indicators included: the share of infants being breastfed at four months post-partum (both exclusively and non-exclusively), tobacco smoke exposure during the first four weeks post-partum (either parent smoking), two-year-olds with complete immunisation coverage for measles, mumps and rubella (two injections) and prevalence of age-adjusted overweight and obesity among four-year-olds (ISO body mass index ⩾25 kg/m^2^).

Seven socio-economic and demographic indicators were compiled for each city district using data from 31 December 2017 obtained from Statistics Sweden. These data consisted of the total population of each district, the share of the population consisting of children 0–17 years old, the share of the population born outside of Sweden or with two parents born abroad, mean income, unemployment rates among adults aged 18–64 years, the share of adults aged 18–64 years without completed secondary education and the share of ninth grade students eligible for upper secondary school.

Data for the indicators were collected for Sweden’s three major cities. These were chosen as geographical units due to availability of data on a city district level, enabling intra-urban comparison, and due to their relatively large populations and hence higher likelihood of collected data being representative for each city district.

### Analysis

Demographic, socio-economic and health data were analysed to generate descriptive statistics for each indicator.

To construct the index, two types of weighting were used. The first weighting consisted of standard deviations in the material calculated for each individual indicator rather than absolute prevalence presented as percentages. This ensured that indicators with large and small percentage variations in the material made similar contributions to the final index value. Furthermore, a principal component analysis of all included indicators was performed, which generated individual factor values for each indicator. These factor values were multiplied with the standard deviations for each data point in the material. All indicators were standardised using the equation:

$$Is\;=\;\left(I\;-\;\frac{M}{S}\right)\;\times \;F$$

where *I* was the indicator value, *M* the mean indicator value, *S* the standard deviation for the indicator, *F* the calculated factor value for the indicator and *Is* the standardised indicator value. The final index values were generated by calculating the mean of the standardised indicator values for each respective city district.

Correlations between index outcomes and socio-economic indicators were calculated using multiple linear regression. All analyses were performed using IBM SPSS Statistics for Windows v26.0 (IBM Corp., Armonk, NY).

### Visualisation

Variations in index values were mapped using Tableau v2018.3.1 (Tableau Software, Seattle, WA) and geospatial vector shapefiles containing data on geographical outlines of administrative city units. The shapefiles were open source and openly available online for Stockholm [23] and Gothenburg [24], and the shapefile for Malmö was obtained from the regional housing and urban development office. To provide enough statistical detail, an older city district division from before July 2013 was used for Malmö, consisting of 10 districts rather than the current five being used after July 2013. A colour palette consisting of negative and positive values represented by different colours was assigned to the geographical units, with darker colours representing lower index values.

## Results

### Demographic, socio-economic and health indicator descriptions

Table II summarises the descriptive statistics for demographic and socio-economic indicators, health indicators and the index outcomes. The data are presented on a city district level, so that each data point represents indicators or index values for a single district. The full data set for all indicators can be found in the Supplemental Material.

**Table II.** Descriptive statistics for indicators and index.

	Indicator	Range	Minimum	Maximum	Mean	*SD*	Variance	Skewness	Factor value
Sociodemographic indicators	Population size	116,494	13,244	129,738	54,479	23,769	564,947,489	0.878	
	Share of population <18 years	18.5%	10.9%	29.4%	20.7%	4.5%	0.2%	−0.168	
	Foreign background	68.6%	19.9%	88.5%	39.1%	19.8%	3.9%	1.095	
	Mean income, SEK/year	290,500	215,000	505,500	347,413	71,585	5,124,364,568	0.468	
	Eligibility for upper high school	41.3%	56.5%	97.8%	83.5%	9.8%	1.0%	−0.75	
	Unemployment	10.2%	1.5%	11.7%	4.6%	2.6%	0.1%	1.123	
	Lacking completed secondary education	25.9%	4.7%	30.6%	13.1%	6.8%	0.5%	0.852	
Health indicators	Tobacco exposure in utero	13.9%	0.1%	14.0%	3.1%	2.8%	0.1%	1.89	0.689
	Low birthweight	3.5%	3.0%	6.6%	4.5%	0.9%	0.0%	0.328	0.606
	Tobacco exposure at 0–4 weeks	33.1%	0.8%	33.9%	12.0%	8.8%	0.8%	0.913	0.85
	Breastfeeding at four months	24.1%	63.10%	87.2%	77.4%	5.9%	0.3%	−0.274	0.667
	Enrolment in daycare or pedagogic care from one to five years	23.8%	67.1%	90.9%	82.5%	4.4%	0.2%	−1.178	0.495
	Vaccination for MMR at two years	11.7%	87.2%	98.9%	96.4%	2.6%	0.1%	−2.21	0.677
	Overweight or obesity at four years	10.6%	5.5%	16.1%	10.3%	2.5%	0.1%	0.437	0.663
Index		4.021	−2.577	1.444	0.032	0.94	0.883	−0.782	

The data presented are on a city district level, so that each data point represents indicator or index values for a single district.

*SD*: standard deviation.

In absolute terms, the highest percentage range for health indicators could be seen for tobacco exposure at 0–4 weeks (0.8–33.9%, standard deviation (*SD*)=8.8%) and breastfeeding at four months (63.1–87.2%, *SD*=5.9%). The largest geographical differences in relative terms were seen for tobacco exposure in utero and tobacco exposure at 0–4 weeks, where the city districts with the lowest and highest prevalence differed by a factor of 140.0 and 42.4, respectively. For demographic and socio-economic indicators, foreign background (19.9–88.5%, *SD*=19.8%) and eligibility for upper high school studies (56.5–97.8%, *SD*=9.8%) had the highest percentage range. Population size and unemployment had the largest differences between city districts in relative terms, differing by a factor of 9.8 and 7.8, respectively.

### Index outcomes

**Figure 1.**
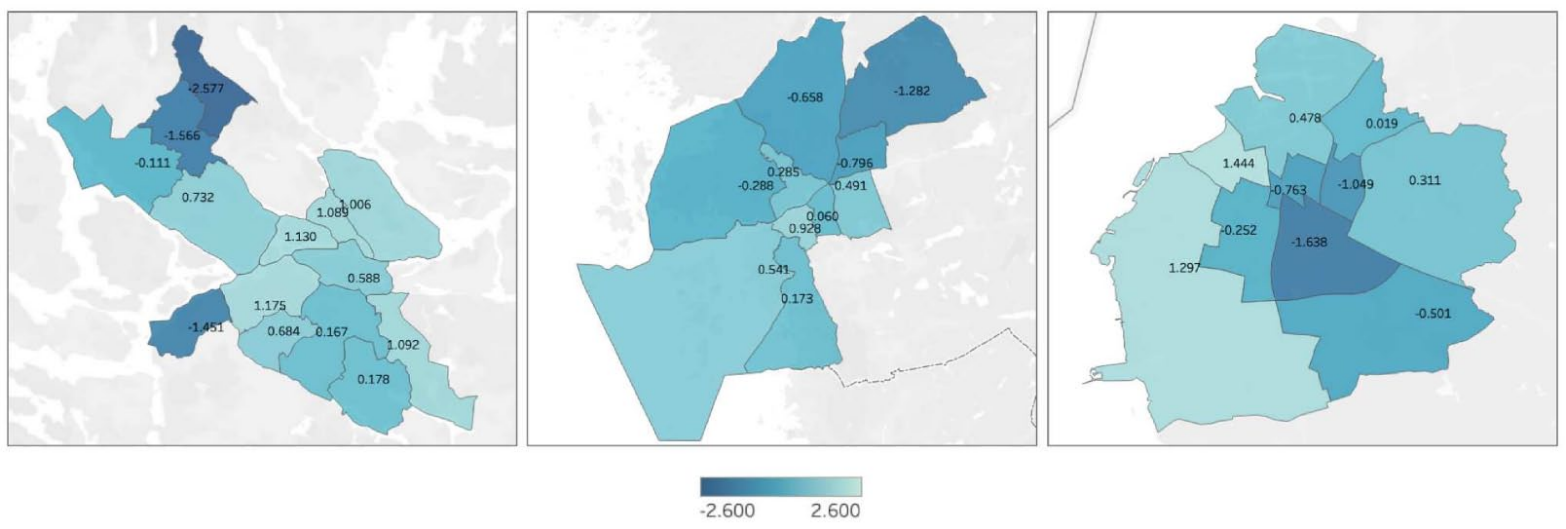
Geographical distribution of child health index outcomes in city districts of Stockholm, Gothenburg and Malmö.

Index values varied from −2.577 to 1.444, with a median of 0.173, as presented in Table II. The index values had a standard deviation of 0.940 and a negative skewness. The resulting map of the geographical distribution of index values is presented in Figure 1. The median index outcome for Stockholm was 0.588, with a variance of −2.577 to 1.175, where the lowest value was seen for the district Rinkeby-Kista. Gothenburg had a median of 0.060, with a variance of −1.282 to 0.928, with the lowest value for the district Angered. The median for Malmö was 0.019, with a variance of −1.638 to 1.444, with the lowest value for the district Fosie.

### Correlations with socio-economic and demographic indicators

Linear regression was used to assess correlations between sociodemographic factors and index scores for districts. All socio-economic factors displayed a significant correlation (*p*<0.05) in the bivariate analysis, and consequently all variables were included in a multivariate analysis. Foreign background and mean income remained significant in the multiple linear regression model, with correlations of *R*^2^=0.364 and *R*^2^=0.185, respectively (Table III).

**Table III.** Multiple linear regression analysis displaying correlations between index outcomes and sociodemographic indicators.

Indicator	Pearson correlation (*R*^2^) to index	*p*-Value
Population size	0.001	0.902
Foreign background	0.364	0.001
Lack of completed secondary education	0.031	0.368
Unemployment	0.001	0.841
Mean income	0.185	0.022
Eligibility for upper high school	0.113	0.080
Share of population <18 years	0.003	0.779

## Discussion

The lives of children growing up in segregated cities are shaped differently by their respective environments. Cities with marked socio-economic inequality thus also display inequities in health on several levels – measured as risk factors, protective factors, health outcomes and utilisation of support. Such disparities can be seen between social groups, but also between geographic areas, as displayed by the index outcomes.

The index showed an aggregation of low values in the north-western and western districts of Stockholm. In Gothenburg, there was a similar gradient along a south-west to north-east axis, with lower index values in the north-east. Malmö displayed lower index values in the central parts of the city, and the highest values in the western parts of the city. All cities had similar geographic trends for indicators of socio-economic status, displaying how the social gradient is geographically distributed in Swedish metropolitan areas.

The city district with the lowest index outcomes was Rinkeby-Kista, an area in north-western Stockholm. As displayed in the Supplemental Material, the district had generally poor values for indicators measuring both protective factors (breastfeeding at four months, 75.1%), risk factors (Tobacco smoke exposure at 0-4 weeks, 18.9%), health outcomes (Overweight and obesity at 4 years, 15.3%) as well as utilisation of support (Children 0-5 years in preschool, 67.1%), illustrating how the outcome for several different types of indicators can follow similar patterns. Stockholm also showed the largest disparities in child health, measured as variance in index outcomes.

Index outcomes were significantly correlated to all socio-economic and demographic indicators in the bivariate analysis, following an apparent social gradient. The significant correlation to foreign background in the multivariate analysis displays how child health in Sweden follows a pattern of ethnic segregation, and points towards an unmet need to improve early life conditions among children in areas with high immigration. The weakest correlation was found between index outcomes and district population size, which was still significant in the bivariate analysis. We theorise that this could potentially be explained by central districts having both higher living standards and higher population density and thus larger populations.

The trends for the index in the three cities is in line with what has been previously reported, such as data for individual indicators in Stockholm and Malmö [15,19]. A report on disparities in child health in Gothenburg, presented by the Nordic School of Public Health in 2013, displayed a similar gradient in the city by compiling indicators without using weights, resulting in indicators with small percentage variations in the data contributing significantly less to variations in the final index outcomes [17]. In contrast to previous reports and research, this paper may provide both an overview of the geographical inequities of several aspects of child health, and allowing both intra- and inter-urban comparison.

Smaller urban areas and municipalities may have similar disparities in child health that ought to be assessed, but the lack of accessible data limits similar intra-urban comparisons in many parts of Sweden. This presents a barrier for generalisability of the index on a national level. The same method for mapping disparities in health used in this paper could also be used in other urban contexts internationally, where other indicators may be more relevant to use due to reasons of epidemiology or data availability. Such indicators may include incidence of child mortality or communicable diseases, as these constitute a higher burden in low- and middle-income regions of the world [25]. A lack of standardisation of data collection (e.g. at what age child health data are recorded) may, however, affect the possibilities for creating an index used for international comparison, which is why this was not set as an aim for this article.

Aggregating data on a city district level may hide variations within these relatively large geographical units. Utilising non-aggregated data or aggregated data on a geographically smaller level would have enabled more detailed analyses, but would also have been more resource intensive. The index covers the four domains of child health that were stipulated as desirable to include. Some indicators that could have provided valuable information on child health were, however, not included in the creation of this index, partly due to lack of standardisation in how the data for the indicators were collected or registered. These included the prevalence of dental caries, measurements of mental health and participation in parental support groups. Other indicators, such as child mortality, could not be broken down at the desired low geographic level due to very low incidence and were thus excluded. Currently, only two of Sweden’s 21 regions are connected to the Swedish Child Preventative Health Registry [26], and few local authorities compile child health data that are accessible through regional offices. Limitations in available data prevent the index from covering every aspect of child health, and future attempts to map child health inequities with this method may have more opportunities for this if national registers receive better coverage.

Our index provides a visualisation of geographical segregation in child health and its correlation to socio-economic indicators in Sweden’s three largest cities, highlighting where health-promoting interventions aimed towards children may be most relevant to implement. As displayed by the large variations in the data and index outcomes, there is currently a pressing need for such initiatives. Future research may want to explore further both the driving mechanisms behind these disparities in child health and how the currently unmet needs can be overcome.

## Conclusions

Large disparities in child health exist in Sweden’s three largest cities. Using data from national data sets combined into a single composite index, the geographical distribution of these inequities was shown to follow distinct social gradients in Stockholm, Gothenburg and Malmö. The largest disparities in child health were seen in Stockholm. The strongest correlation between index outcomes and socio-economic and demographic indicators was to foreign background, displaying how children’s health in Sweden follows a pattern of ethnic segregation. The outcomes thus visualise the social gradient in Swedish cities and its effect on children’s health, and displays where initiatives to improve child health may be most relevant to undertake.

## Supplemental Material

sj-xlsx-1-sjp-10.1177_1403494820980261 – Supplemental material for Using an urban child health index to detect intra-urban disparities in Sweden Click here for additional data file. Supplemental material, sj-xlsx-1-sjp-10.1177_1403494820980261 for Using an urban child health index to detect intra-urban disparities in Sweden by Per Kåks and Mats Målqvist in Scandinavian Journal of Public Health
